# Antiviral Activity of Zinc Finger Antiviral Protein (ZAP) in Different Virus Families

**DOI:** 10.3390/pathogens12121461

**Published:** 2023-12-17

**Authors:** Kívia Queiroz de Andrade, Claudio Cesar Cirne-Santos

**Affiliations:** 1 Laboratory of Immunology of Infectious Disease, Immunology Department, Institute of Biomedical Sciences, University of São Paulo, São Paulo 05508-000, SP, Brazil; 2 Laboratory of Molecular Virology and Marine Biotechnology, Department of Cellular and Molecular Biology, Institute of Biology, Federal Fluminense University, Niterói 24020-150, RJ, Brazil

**Keywords:** ZAP protein, PARP-13, antiviral activity

## Abstract

The CCCH-type zinc finger antiviral protein (ZAP) in humans, specifically isoforms ZAP-L and ZAP-S, is a crucial component of the cell’s intrinsic immune response. ZAP acts as a post-transcriptional RNA restriction factor, exhibiting its activity during infections caused by retroviruses and alphaviruses. Its function involves binding to CpG (cytosine-phosphate-guanine) dinucleotide sequences present in viral RNA, thereby directing it towards degradation. Since vertebrate cells have a suppressed frequency of CpG dinucleotides, ZAP is capable of distinguishing foreign genetic elements. The expression of ZAP leads to the reduction of viral replication and impedes the assembly of new virus particles. However, the specific mechanisms underlying these effects have yet to be fully understood. Several questions regarding ZAP’s mechanism of action remain unanswered, including the impact of CpG dinucleotide quantity on ZAP’s activity, whether this sequence is solely required for the binding between ZAP and viral RNA, and whether the recruitment of cofactors is dependent on cell type, among others. This review aims to integrate the findings from studies that elucidate ZAP’s antiviral role in various viral infections, discuss gaps that need to be filled through further studies, and shed light on new potential targets for therapeutic intervention.

## 1. Introduction

Organisms have several ways of sensing and controlling viral infections. This recognition occurs mainly through the detection of viral ribonucleic acid (RNA) or deoxyribonucleic acid (DNA) [1]. This triggers intracellular signaling events that ultimately result in the production of antiviral molecules [2]. In order for the virus to replicate successfully, it is crucial to evade the immune response of the host cell. As a result, the cell develops various recognition mechanisms and restriction factors to control infection. One of these restriction mechanisms is the degradation of viral RNA (vRNA). There are several cell intrinsic antiviral proteins that bind to RNA, regulate translation, and target it for decay, thereby interfering with different stages of the virus replication cycle [3]. Some of these restriction factors are induced by type I interferons [4], which create an antiviral state in neighboring cells. The human CCCH-type zinc finger antiviral protein (ZAP) acts as a post-transcriptional RNA restriction factor in the host cell for viruses such as retroviruses [5], filoviruses [6], alphaviruses [7]. ZAP also inhibits RNA translation and targets the vRNA for degradation [8,9,10]. ZAP is considered an interferon (IFN)-stimulated gene (ISG) that can be induced by viral infection [11]. It is capable of restricting several negative-sense single-stranded RNA viruses [12] and positive-sense single-stranded RNA viruses [9]; however, it is unclear what influence ZAP has on double-stranded RNA (dsRNA) viruses and other higher-order structured RNAs. ZAP was discovered as a protein with antiviral activity in rat cells that showed resistance to Moloney murine leukemia virus (MoMuLV or MMLV) infection [13]. In the presence of the ZAP protein, some viruses, such as herpes simplex virus type 1 (HSV-1), yellow fever virus (YFV) [7], Zika virus (ZIKV), and dengue virus (DENV) [10], are able to grow normally. Interestingly, this ability is not dependent on belonging to the same family, as coxsackievirus B3 [14] but not poliovirus [7], both from the Picornaviridae family, was inhibited by ZAP.

There are four isoforms of ZAP: the short isoform ZAP (ZAP-S), the medium isoform ZAP (ZAP-M), the long isoform ZAP (ZAP-L), and the extra-long isoform ZAP (ZAP-XL), with ZAP-S and ZAP-L being the most prevalent isoforms [15,16]. ZAP-L contains a poly(ADP-ribose) polymerase (PARP) domain and a CaaX motif, which undergoes S-farnesylation. This S-farnesylation is necessary for its antiviral activity against some viruses, such as Sindbis virus (SINV) [17,18]. This S-farnesylation appears to be the reason why ZAP-L is found in plasma membranes or membranous compartments (such as endolysosomes and endoplasmic reticulum) inside cells, while ZAP-S is found in the cytoplasm [17,18].

It is known that ZAP binds to the CpG (cytosine-phosphate-guanine) dinucleotide sequence of viral RNA [19], directing it to the degradation pathway with the help of cofactors such as Tripartite Motif Containing 25 (TRIM25) [20,21]. This leads to the inhibition of viral replication and the assembly of new virus particles [22,23]. The ZAP protein also plays a role in the final stages of virus replication through mechanisms that have yet to be determined [24,25]. The specificity of the binding between CpG dinucleotide and vRNA still needs to be better understood. Additionally, some studies suggest that the ZAP protein also exhibits antiviral activity by binding to UpA dinucleotides in certain viruses, thereby attenuating their replication. However, further research is needed to confirm whether ZAP directly binds to UpA dinucleotides [26,27].

The CpG sequence can potentially be used for the development of viral therapies in order to prevent diseases caused by viral infections. In this review, we will discuss the current understanding of the mechanisms employed by ZAP to inhibit viral replication.

## 2. Zinc Finger Antiviral Protein—ZAP

The zinc finger antiviral protein (ZAP), also known as Poly(ADP-ribose) Polymerase-13 (PARP-13), ADP-Ribosyl-Transferases Diphtheria Toxin-Like-13 (ARTD13), Zinc finger CCCH-type and antiviral 1, is encoded by the human gene ZC3HAV1 (zinc finger CCCH-type containing, antiviral 1, Chromosome 7). ZAP belongs to the PARP protein family (poly(ADP-ribose) polymerase), which uses nicotinamide adenine dinucleotide (NAD^+^) as a substrate to generate modifications in acceptor proteins but lacks poly(ADP-ribosylation) activity [13,28,29].

ZAP plays a role in the intracellular host cell’s immune system by detecting positive and negative single-stranded RNA viruses from various families during infection. However, it does not bind to double-stranded RNA viruses (dsRNA) [29]. ZAP exhibits antiviral properties by directing these viruses to degradation pathways and/or inhibiting their translation, thus restricting viral replication. This prevents the accumulation of viral RNA in the cytoplasm and hinders the virus from multiplying. The viruses affected by ZAP include murine leukemia virus (MLV), Sindbis virus (SINV), and the RNA intermediate of the hepatitis B DNA virus, among others (Table 1) [6,7,13,28,30,31,32,33].

The presence of orthologs in certain animals, such as mammals, fish, and reptiles, indicates that ZAP has a distant origin [15,34]. A phylogenetic analysis revealed that the ZAP gene originated in tetrapods [35]. Gongalves-Carneiro et al. (2021) [35] tested ZAP-related proteins from tetrapods and found that these proteins have an antiviral role in human cells [35]. ZAP was initially described in Rat2 cells, where it reduced the replication of MMLV [13] and later in humans [15].

ZAP is consistently expressed in human cells (hZAP) and has two main isoforms derived from alternative splicing. The long isoform, known as ZAP-L or PARP-13.1, consists of 902 amino acids and is associated with the membrane (it contains the YYV catalytic motif). The short isoform, called ZAP-S or PARP13.2, consists of 699 amino acids and is located in the cytosol. Both isoforms originate from the same exon [15,28,36]. Li et al. (2019) [16] discovered two additional splice variants of ZAP in humans: ZAP-XL (extralong) and ZAP-M (medium). However, the ZAP-L and ZAP-S isoforms are the most abundant [15,16]. Furthermore, they found that the longer isoforms (ZAP-L and ZAP-XL) have a greater impact on alphavirus and hepatitis B virus (HBV) (DNA virus) compared to ZAP-S and ZAP-M [16]. ZAP can be activated by viral infection due to the presence of binding sites for the signal transducer and activator of transcription (STAT) and Interferon Regulatory Factor 3 (IRF3) in its promoter [11,37,38,39]. ZAP-L is constitutively expressed in Huh7 cells and acts promptly upon infection, whereas ZAP-S is expressed dependent on IFN signaling [17,18,28,31,40,41,42]. Okudera et al. (2022) [43] found that treatment of human cells with polyinosinic-polycytidylic acid (poly IC), a Toll-like receptor 3 (TLR3) agonist, upregulated ZAP-S expression. However, when cells were transfected with siRNA against IRF3 or siRNA against TRIM25, the upregulation of ZAP-S was reduced upon treatment with poly-IC [43]. The mechanisms through which IRF3 and TRIM25 regulate ZAP-S expression need further clarification.

**Figure 1:**
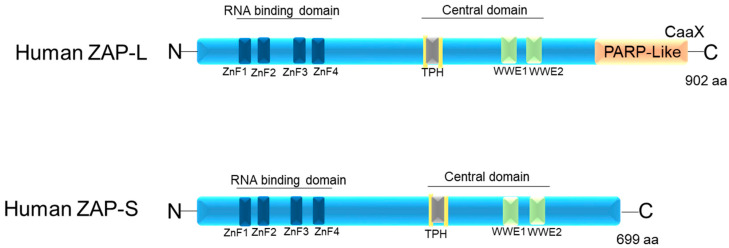
Schematic image of the protein domains of the two isoforms of human ZAP: ZAP-L and ZAP-S. ZnF1–4: four CCCH-type zinc finger motifs. TPH (or TiPARP Homology domain (conserved among ZAP paralogs and containing a fifth CCCH zinc finger motif). WWE motifs. CaaX: C is cysteine, A is usually two aliphatic amino acids, and X can be a variety of amino acids.

ZAP in humans has three structural domains: (1) an N-terminal RNA-binding domain (RBD) (amino acids [aa] 1–240) that has four CCCH-type zinc fingers; (2) an integrated central domain (aa 241–700) that contains a TPH domain (TiPARP homology region) containing a fifth zinc finger motif and two WWE modules (Domain in Deltex and TRIP12 homologues (Thyroid Hormone Receptor Interactor 12)); and (3) catalytically inactive C-terminal poly(ADP-ribose) polymerase (PARP)-like domain (aa 701–902) with a regulatory function [15,25,35,44,45] (Figure 1). According to Kerns et al. (2008) [15], although ZAP-S has a broader expression pattern than ZAP-L, expression of ZAP-L was observed in cell lymphocytes and germline tissues [15].

The C-terminal poly(ADP-ribose) polymerase (PARP)-like domain is absent in ZAP-S (PARP-13.2 isoform) and enzymatically inactive in ZAP-L, as it lacks the histidine, tyrosine, and glutamate (H-Y-E) catalytic triad [46]. However, all isoforms contain both the N-terminal and central domains [13,30,47,48] (Figure 1). The targeting of ZAP-L to mem-branes is possible due to the presence of the PARP domain that contains a cysteine (CaaX) motif, which mediates S-farnesylation [18] (Figure 1). This explains its greater presence in vesicular compartments and the cytoplasmic membrane inside the cell, while ZAP-S is mainly found in the cytoplasm [17,40]. The hypothesis is that ZAP-L can inhibit viruses whose entry into the cell occurs through endocytosis. Furthermore, it may have antiviral activity against viruses that replicate in membrane-derived compartments [49,50]. Another hypothesis is that ZAP-L is targeted to membranes to form antiviral complexes with its cofactors to exert its antiviral activity. This hypothesis is the most accepted, as ZAP-L targets vRNAs that have several replication sites and mechanisms [15,16,31,45,46,51,52].

It is not yet well established whether the two isoforms (ZAP-L and ZAP-S) of ZAP in humans have different roles or overlap in antiviral activity. ZAP-L is known to inhibit alphaviruses and HBV better than ZAP-S [16]. Although both isoforms have the N-terminal domain, they seem to restrict viruses in different ways, as we will discuss later.

A mutation in the cysteine 88 to arginine (ZAPC88R) in the zinc fingers domain of ZAP has been observed to result in the complete loss of antiviral activity [53]. The interference of the second (ZnF2) and fourth zinc finger (ZnF4) motifs appears to inhibit the antiviral activity of ZAP, while mutations in other ZnF motifs have a milder impact on the protein’s function [19,30]. Meagher et al. (2019) [54] showed that the ZnF2 motif contains a pocket that selectively packs CG dinucleotides, while ZnF3 contains a binding pocket for guanine and cytosine and ZnF4 for cytosine [19,54]. ZAP forms a dimer that binds to ZAP-responsive element (ZRE) sequences in the target viral RNA, and these ZREs are specific to ZAP [44,48,55] (Figure 1). Yang et al. (2022) [9] found that mutations affecting the binding of both ZAP and TRIM25 to RNA interfere with antiviral activity against Sindbis virus (SINV). They discovered that mutations in the ZnF of both ZAP-S (mutation in ZnF1 or ZnF3) and ZAP-L (mutation in ZnF1) affected SINV RNA binding. Furthermore, the mutation in ZnF4 of ZAP-L increased SINV replication, but mutations in ZnF from ZAP-S did not, possibly due to the greater antiviral activity of ZAP-L compared to ZAP-S [9].

## 3. RNA Recognition by ZAP

The target mRNA region to which the ZAP protein binds is rich in CpG (cytosine-phosphate-guanine) dinucleotides, which are two adjacent nucleotides in a linear sequence. After binding, a decrease in vRNA in the cytoplasm is observed, indicating degradation of RNA or translational repression [22]. Each ZAP molecule binds to a CpG, allowing it to form an oligomer in the target viral RNA [19,48,54]. This specificity is due to an integrated pocket that accommodates only CpG dinucleotide sequences and is contained within a larger RNA-binding domain [11,14,51].

In vertebrate genomes, the cytosine of a CpG dinucleotide is prone to methylation. This is due to the presence of DNA methyltransferases, enzymes that catalyze the conversion of cytosines in a CpG context to 5-methylcytosine. Methylation is followed by deamination and mutation, resulting in the gradual replacement of CpG dinucleotides by TpG and CpA [56]. However, CpGs in RNA viruses are not subject to the same methylation and mutation pressure. Therefore, ZAP has little effect on human mRNA, as vertebrate cells have a suppressed CpG dinucleotide frequency [57]. Some viruses have evolved genomes that are suppressed by CpG-enriched segments, avoiding detection by ZAP [22]. Based on this, some viruses can modify their CpG dinucleotides in order to develop live attenuated vaccines. Other studies have demonstrated that ZAP detection of foreign genomes also involves RNA secondary structures containing stem loops with conserved sequences, such as “GGGUGG” and “GAGGG” [58]. Altering this conserved region leads to diminished RNA recognition and a decrease in ZAP’s antiviral function [58]. It is worth noting that ZAP sensitivity to alphaviruses does not correlate with CpG dinucleotide content [16]. Further analysis is needed to determine whether ZAP binding to a specific region of alphavirus RNA is due to structural reasons or linear sequence motifs. Nguyen et al. (2023) [59] found that in the genome of ZAP-sensitive alphaviruses (Ross River virus (RRV), Sindbis virus (SINV)), there were three 500 bp sequences correlated with CpG in the non-structural protein (nsP) gene region, compared to ZAP-resistant alphaviruses (o’nyong’nyong virus (ONNV), chikungunya virus (CHIKV)). Furthermore, the nsP2 region of ZAP-sensitive alphaviruses is crucial for ZAP sensitivity, and its binding is CpG-dependent [59].

Additionally, viruses with increased CpG dinucleotide content do not always guarantee sensitivity to ZAP. Studies have reported that ZAP is able to restrict viruses with abundant UpA (uracil-phosphate-adenine) dinucleotides [16,26,27,45,60]. Further research is needed to demonstrate the binding of ZAP to the UpA sequence, the mechanisms behind this binding, and its antiviral role. Gonçalves-Carneiro et al. (2022) [61] found that the number, spacing, and surrounding sequence of CpG dinucleotides in the env gene are important for ZAP sensitivity. With this understanding, it is possible to study the generation of a mutant virus genome with modifications that act as a live attenuated vaccine and are precisely prevented by the ZAP protein, as proposed by the author [61]. ZAP can bind to RNA with a low frequency of CpG dinucleotides, as the location of these CpG motifs is crucial for these viruses to be restricted [51,52]. This demonstrates that key points in the understanding of ZAP’s restriction of vRNA still need to be elucidated.

## 4. Cofactors Required by ZAP for its Antiviral Activity

Given that ZAP lacks RNase activity, it relies on other mechanisms for its antiviral activity. One such mechanism involves its interaction with a cellular polyadenylate-specific ribonuclease (PARN), which degrades the poly(A) tail. ZAP can also recruit an exosomal complex that contains exoribonucleases with 3’-5’ activity, such as ribosomal RNA-processing protein 46/exosome complex component (RRP46/EXOSC5) and ribosomal RNA-processing protein 42/exosome complex component (RRP42/EXOSC7), resulting in the cleavage of viral RNAs [55,62]. Elements bound to ZAP can also engage the decapping complex (decapping protein 1 (DCP1) and DCP2) through the RNA helicase p72 (DDX17), leading to the removal of the 5’ cap structure of the mRNA. Additionally, the 5’-3’ Exoribonuclease 1 (XRN1) is involved in the process of viral RNA degradation [55]. Further studies are needed to investigate the interaction between ZAP isoforms and these proteins with antiviral activities to determine whether there is a preference in the interaction of these proteins with ZAP-L or ZAP-S.

It has also been suggested that ZAP can be observed in cytoplasmic RNA stress granules, even in the absence of viral infection. These stress granules determine the fate of mRNAs that are not involved in translation, either stabilizing them or directing them towards degradation pathways. This suggests that ZAP may be involved in the regulation of cellular mRNA [36,41].

Since ZAP lacks nuclease activity, it recruits proteins such as TRIM25 to act as cofactors in mediating its antiviral activity [20,21,63]. It is important to identify all the accessory proteins, including those with endonuclease domains, that interact with the N-terminal domain of ZAP to better understand its mechanism of inhibiting viral replication. This highlights the involvement of other host factors that contribute to the innate immune response in addition to the ZAP protein. To gain a better understanding of ZAP’s antiviral activity and its mechanism of action, it is crucial to describe these cofactors in more detail.

### 4.1. Tripartite Motif Containing 25 (TRIM25)

TRIM25 is a member of the tripartite motif (TRIM) family of proteins, which play a role in supporting the host’s innate immune response to viral infections [64]. It is an E3 ubiquitin ligase enzyme that is induced by type I IFN [65]. The protein consists of an N-terminal RING domain, followed by a B-box type 1 domain, a B-box type 2 domain, a coiled-coil domain (CCD), and a C-terminal SPRY domain [64]. TRIM25 has the ability to bind to RNA, which is crucial for its subcellular localization and antiviral activity [66]. Additionally, TRIM25 can modify ZAP-S and ZAP-L through K48- and K63-linked polyubiquitin [20].

**Figure 2:**
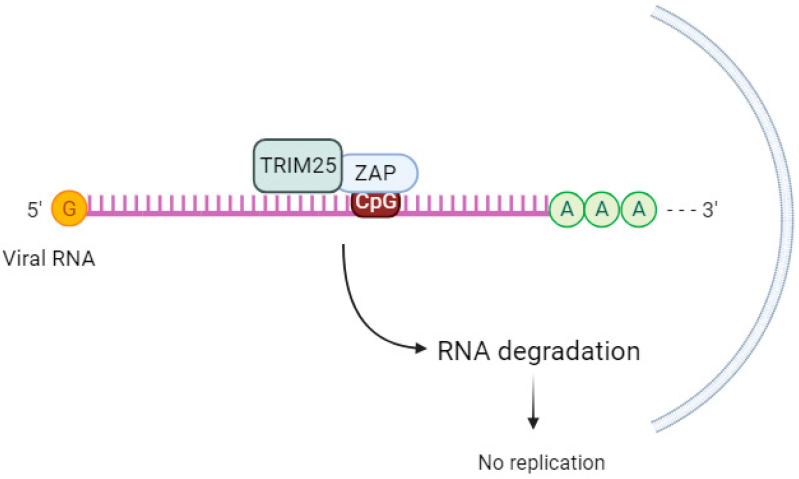
TRIM25 acts as a cofactor of ZAP. ZAP binds to the CpG sequence in viral RNA and, upon sequential binding of TRIM25 and catalytic activation, induces downstream signaling to inhibit viral replication.

TRIM25 enhances the antiviral activity of ZAP (Figure 2), but the mechanism behind this cooperation is not well understood. While TRIM25 can bind to both single-stranded and double-stranded RNA [66], the involvement of ZAP in this process is not fully understood. ZAP and TRIM25 are both interferon-stimulated genes in human cells [67]. According to Li et al. (2017) [20], TRIM25 lacking the RING domain or coiled-coil domain loses its ability to stimulate ZAP’s antiviral function [20].

The ubiquitination of ZAP by TRIM25 does not seem to be crucial for ZAP’s antiviral activity. When Li et al. (2017) [20] used a ZAP-S 7UbΔ mutant that cannot be ubiquitinated, there was still inhibition of SINV replication. This suggests that other factors are involved in TRIM25-mediated upregulation of ZAP’s antiviral activity. The same group discovered that in the absence of TRIM25, ZAP-mediated SINV translation was reduced [20]. Other authors [68] propose that TRIM25-mediated ubiquitination of other substrates leads to the activation of the antiviral state [68].

Gonçalves-Carneiro et al. (2021) [35] observed that the functional interaction between TRIM25 and ZAP is inherently protein–protein via the N-terminal ZAP zinc finger domain and the C-terminal SPRY domain of TRIM25, and this interaction is not RNA-dependent. A hypervariable sequence in the N-terminal domain of ZAP was important for the species-specific dependence of the ZAP protein on TRIM25 [35,69].

In addition to acting as a cofactor of ZAP, TRIM25 can also affect the expression of both ZAP isoforms by regulating alternative splicing, which is necessary for effective ZAP-S expression [70]. Yang et al. (2022) [9] demonstrated that the ZAP and TRIM25 interaction acts to inhibit the translation of Japanese encephalitis virus (JEV) in 293T cells transfected with replication-defective JEV replicon RNA reporter and subsequently transfected with a reporter gene to measure luciferase activity [9]. Further studies are needed to clarify the downstream mechanisms after TRIM25 and ZAP interaction, as well as the impact of ZAP ubiquitination on its antiviral activity. Furthermore, studies are needed to verify in what viral contexts TRIM25 regulates ZAP expression.

### 4.2. KH and NYN Domain Containing (KHNYN)

**Figure 3:**
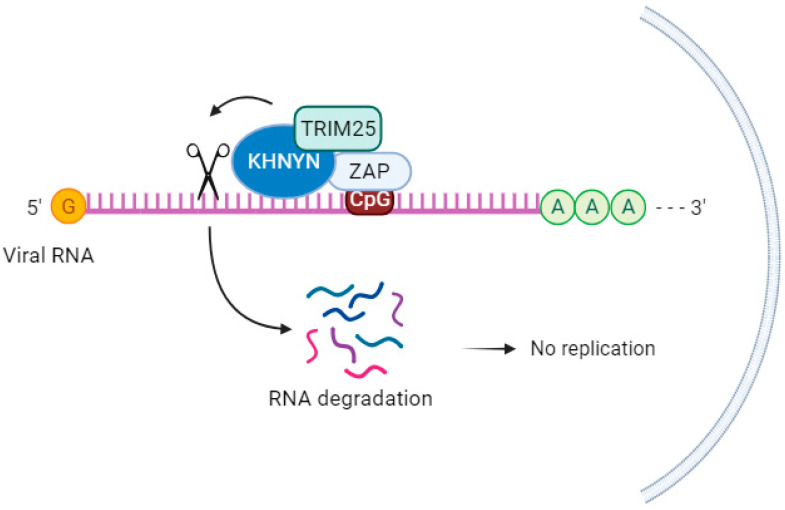
KHNYN acts as a cofactor for the antiviral activity of ZAP. KHNYN interaction with ZAP is important for the inhibition of HIV-1 containing clustered CpG dinucleotides, and this inhibition requires TRIM25.

KHNYN, a cytoplasmic protein containing an NYN ribonuclease domain, has been identified as an accessory protein for the antiviral activity of ZAP. It targets viral RNAs for degradation [71]. In cells lacking ZAP but expressing high levels of KHNYN, there was no substantial inhibition of genomic RNA (gRNA) abundance of HIV-1 with CpGs introduced in the Env protein (HIV-1EnvCpG86-561). However, in the absence of KHNYN, viruses multiplied more successfully, demonstrating that KHNYN decreases HIV-1 RNA containing CpG dinucleotides in a ZAP-dependent manner. The authors also observed through the immunoprecipitation assay that KHNYN interacts with ZAP-S and ZAP-L. The KH-like domain and NYN domain endonuclease are required for the antiviral activity of KHNYN. Furthermore, KHNYN decreases Gag and Env expression and virion production. The same group also found that the KHNYN interaction with ZAP is important for the inhibition of HIV-1 containing clustered CpG dinucleotides, and this inhibition requires TRIM25. ZAP and KHNYN can directly interact to form a heterodimer, but TRIM25 is not required for this interaction [71] (Figure 3). ZAP targets KHNYN to CpG dinucleotides in viral RNA for cleavage [71]. Kmiec et al. (2021) [25] demonstrated that the PARP domain containing the CaaX motif in ZAP-L is essential for a more efficient interaction with KHNYN and TRIM25 compared to the ZAP-S interaction with these two cofactors. When ZAP-S received the CaaX box, there was a benefit in the interaction with the cofactors, whereas when the CaaX box was mutated, the viral restriction by ZAP-L was overturned [25]. Additionally, they observed that the PARP and CaaX box were essential for antiviral activity against CpG-enriched HIV-1 and SARS-CoV-2. The vesicular localization of ZAP-L seems to correlate with its antiviral action against CpG-enriched HIV-1, and S-farnesylation for ZAP-L was important to inhibit SARS-CoV-2 since this virus replicates in compartments originating from the endoplasmic reticulum (ER) [25].

Further studies are needed to assess how TRIM25 is induced for the interaction between KHNYN and ZAP, the downstream mechanisms after this interaction on viral RNA, and the viral context in which this occurs.

### 4.3. Exosome

The exosome complex contains a 3’-5’ exoribonuclease activity that contributes to the processing and/or degradation of RNA molecules. It is a multisubunit complex that, in humans, has a core containing nine subunits. This includes six proteins with the PH RNase domain (hRrp41p, hRrp42p, hRrp43p, hRrp46p, PM/Scl-75, and Mtr3), as well as three RNA-binding proteins (Rrp40, Rrp4, and Csl4). There are both nuclear and cytoplasmic forms of the exosome, but it is in the cytoplasm where the degradation of mRNAs containing AU-rich elements (AREs) within their 3’ untranslated regions occurs [62]. ZAP targets ZRE-containing mRNAs but not ARE mRNAs [30]. When ZAP binds to the ZRE-containing mRNA, it can require the RNA-processing exosome complex [62]. The N-terminal domain of human ZAP, which contains the exosome-interacting domains, interacts with the exosome component hRrp42 [72]. Inhibition of this component leads to a reduction in the action of ZAP [55]. Guo et al. (2007) [62] found that ZAP binds to the C-terminal fragment of hRrp46p and that depletion of hRrp46p leads to a decrease in ZAP activity [62]. The initiating step of the 3’-5’ decay pathway is the removal of the Poly-A tail by Poly(A) ribonuclease (PARN) [55], followed by degradation of the mRNA from the 5’-end by 5’-3’ exoribonucleases (XRNs) or from the 3’-end [73]. The interaction between the exosome and ZAP was observed by immunoprecipitation assay [74,75].

More studies are needed to verify in which context this exosome recruitment occurs by ZAP in the presence of other cofactors.

### 4.4. p72 RNA Helicase

**Figure 4:**
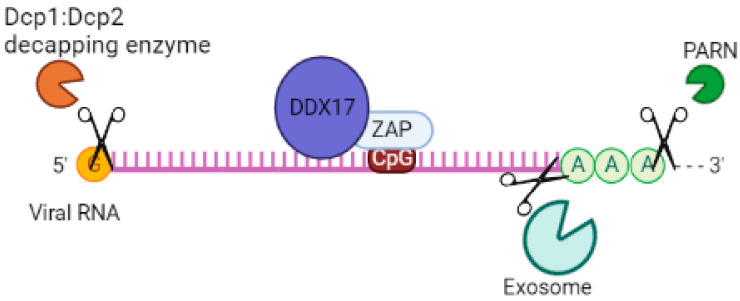
System of cofactors required by ZAP to target viral RNA for degradation. DDX17 increases the efficiency of ZAP by inhibiting virus replication by targeting mRNAs for degradation via the exosome and recruitment of Dcp1:Dcp2 decapping enzyme to the 5′-end of viral RNAs, inhibiting cap-dependent mRNA translation initiation. ZAP interacts with a cellular polyadenylate-specific ribonuclease (PARN), which is a 3’-exoribonuclease that removes poly(A) tails from the 3’ end of RNAs.

p72 RNA helicase, also known as p72 DEAD-box RNA helicase or DDX17 [76,77], is involved in the regulation of RNA structure. It contains a conserved motif Asp-Glu-Ala-Asp (DEAD) and is responsible for ATP-dependent RNA helicase activity. This helicase catalyzes the rearrangement of RNA structure and plays a role in various metabolic processes, including transcription [78], translation, RNA degradation [79], and pre-mRNA processing/alternative splicing [80]. In a study by Chen et al. (2008), it was found that both the N- and C-terminal domains of p72 RNA helicase bind to ZAP, a protein linked to mRNA, in an RNA-independent manner [44]. This interaction enhances the efficiency of ZAP in inhibiting virus replication by targeting mRNAs for degradation through the exosome. Additionally, p72 RNA helicase recruits the Dcp1:Dcp2 decapping enzyme to the 5’-end of viral RNAs, inhibiting cap-dependent mRNA translation initiation and inducing viral RNA degradation. It also recruits the complex exoribonuclease XRN1 [55,81] (Figure 4). Although p72 RNA helicase does not directly interact with the exosome [81], it forms an antiviral complex with it when recruited by ZAP. When p72 RNA helicase is depleted using siRNA, ZAP’s antiviral activity is decreased. Further studies are needed to determine which isoforms of ZAP p72 RNA helicase enhance its efficiency against viruses.

## 5. ZAP Protein Regulators

### 5.1. Matrin 3 (MATR3)

Matrin 3 (MATR3) is an inner nuclear matrix protein that binds to DNA and RNA [82]. It plays a role in various processes, including DNA replication/repair, transcription, and RNA processing [83]. MATR3 possesses two RNA recognition motifs (RRM) that enable it to bind RNA. It has been observed to be part of a protein–RNA complex involved in stabilizing mRNA [84]. MATR3 interacts with DDX17, EXOSC3 (a core component of the human RNA exosome complex responsible for 3’-5’ exoribonuclease activity), and ZAP. When MATR3 is suppressed, ZAP-induced degradation of HIV-1 and MMLV transcripts increases. Consequently, an upregulation of MATR3 expression leads to the downregulation of ZAP, inhibiting its activity and acting as a negative regulator [85]. Further research is necessary to determine whether viruses actively stimulate Matrin 3 protein as an escape mechanism against the immune response through ZAP and its cofactors.

### 5.2. Glycogen Synthase Kinase 3β (GSK3β)

GSK3 is an active protein that lacks typical kinase characteristics. In order for it to function and maintain appropriate levels, localized regulatory mechanisms are necessary. GSK3 substrates typically require prior phosphorylation by another kinase [86]. There are two isoforms of GSK3, GSK3α and GSK3β, which are encoded by separate genes. GSK3β, a serine/threonine protein kinase, has over 500 substrates [86,87]. GSK3 can be found in the nucleus, mitochondria, and mainly the cytosol [86]. In rats, GSK3β phosphorylates the serine residues in ZAP, which is crucial for its optimal antiviral activity against MMLV and HIV-1 pseudovirus. When GSK3β is inhibited or downregulated, the activity of rat ZAP is reduced. Furthermore, the phosphorylation of rat ZAP by GSK3β negatively affects the translation of target mRNA but not its levels [88]. Colmant et al. (2021) [23] discovered that inhibiting the phosphorylation of human ZAP using the kinase inhibitor C16 resulted in a decrease in the expression of protein E from Binjari virus. This virus is an insect-specific flavivirus lineage II that contains a high frequency of CpG dinucleotides [23]. Other studies have shown that the attenuation of CpG-high echovirus 7 (E7), independent of protein kinase RNA-activated (PKR), can be reversed by using the C16 kinase inhibitor [26,89]. Pretreatment with a specific GSK3 inhibitor, SB 216763, increased the replication of CpG-high E7 virus, indicating that the phosphorylation of human ZAP is important for inhibiting CpG-rich viruses [27].

Further studies are needed to understand the mechanisms involved in the regulation of ZAP’s antiviral activity through phosphorylation by GSK3β. Additionally, it is important to verify the reproducibility of these results through in vivo experiments.

## 6. ZAP Inhibits Target Virus RNA Translation

**Figure 5:**
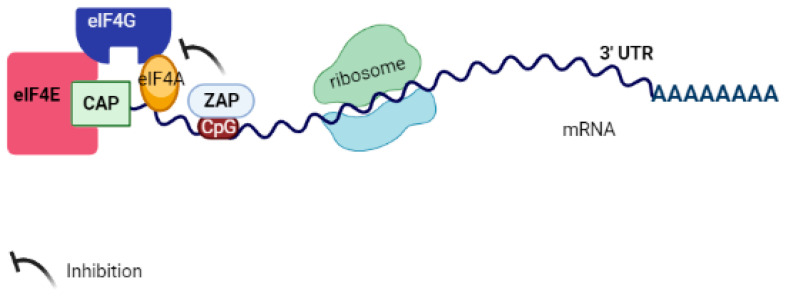
ZAP inhibits viral mRNA translation by inhibiting the formation of eIF4F complex. ZAP, when interacting with initiation factor eIF4A, avoids viral translation by preventing the formation of the eIF4F complex (eIF4E cap-binding protein, eIF4A DEAD box RNA helicase, and eIF4G scaffolding protein). CAP: a structure found at the 5’ end of mRNA.

One of ZAP’s mechanisms of action is inhibiting the translation of viral RNA. For instance, ZAP can impede the translation of Sindbis virus mRNA by interacting with eukaryotic initiation factors (eIFs) involved in translation, such as eukaryotic initiation factor-4A (eIF4A). eIF4A belongs to the DEAD box protein family, which is essential for decoding mRNA. When ZAP binds to eIFs, it hinders the formation of the eIF4F complex. This complex consists of eIF4E cap-binding protein, eIF4A DEAD box RNA helicase, and eIF4G scaffolding protein, ultimately resulting in the blockage of translation [8] (refer to Figure 5). Moreover, other studies have demonstrated that ZAP not only affects viral mRNA but also regulates the expression of cellular mRNA. Additionally, the activity of different isoforms of ZAP varies depending on the transcript [17,36,41,42]. It is crucial to comprehend why ZAP affects the RNA degradation of certain viruses while inhibiting the translation of others. Furthermore, it is important to determine whether translation inhibition is a prerequisite for ZAP-mediated mRNA degradation, which may vary depending on the specific viral context.

## 7. Immune Pathways Associated with Antiviral Activity of ZAP

### 7.1. Type I and III Interferons (IFNs)

Viruses can be recognized by surface pattern recognition receptors (PRRs), such as Toll-like receptors (TLRs), and cytosolic receptors, such as acid-inducible gene I retinoic receptors (RIG-I), also known as DDX58. These receptors stimulate the downstream signaling cascade. When viruses activate TLRs, adapter molecules like myeloid differentiation primary response 88 (MyD88), MyD88 adapter-like (Mal), TIR-domain-containing adapter-inducing interferon-β (TRIF), and TRIF-related adaptor molecule (TRAM) activate transcription factors, including NF-κB, Interferon Regulatory Factor 3 (IRF-3), and Interferon Regulatory Factor 7 (IRF-7). This leads to the expression of proinflammatory cytokines and interferons (IFNs). IFN mRNAs are translated into type I IFN (IFN-I) (IFN-β, 13 subtypes of IFN-α, IFN-ε, IFN-ω, IFN- δ, IFN- τ, and IFN- κ), type II IFN (IFN-II) (IFN-γ), and type III IFN (IFN-III) (IFN-λ1, IFN-λ2, IFN-λ3, and IFN-λ4). Each type is classified according to the receptor it signals through [90,91,92].

**Figure 6:**
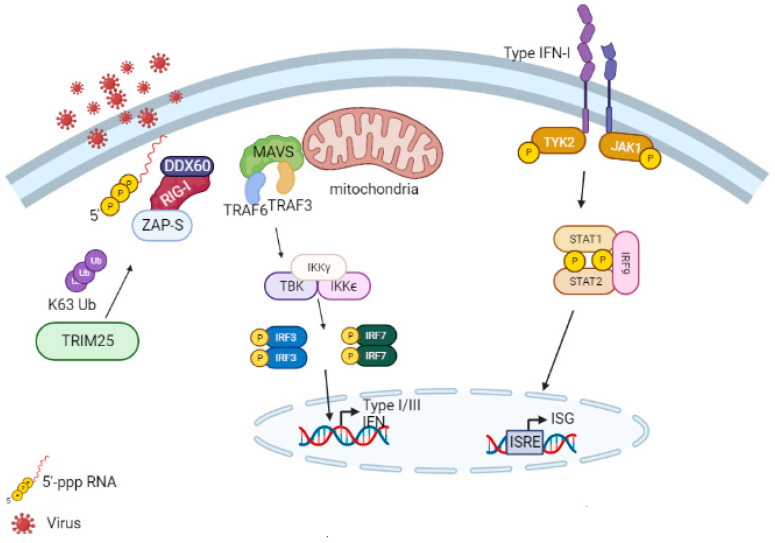
Interaction between ZAP, RIG-I, and IFN in the antiviral immune response: ZAP-S interacts with RIG-I to enhance the oligomerization and ATPase activity of RIG-I. This, in turn, increases the activation of IRF3 downstream when the RIG-I ligand, 3′pRNA, is present in human cells. DDX60 associates with RIG-I and is involved in RIG-I-dependent type I IFN production in response to viral RNA. TRIM25 activates the RIG-I pathway by facilitating K63-linked polyubiquitin chain formation through its E3 ubiquitin ligase activity. This ubiquitination promotes interaction with MAVS, leading to downstream signaling (Crosse et al., 2018 [93]; Martín-Vicente et al., 2017 [64]). DDX60 is a DEXD/H box helicase.

IFN-I and -III signaling activate the JAK (Janus kinase)/STAT (signal transducer and activator of transcription) pathway in an autocrine and paracrine manner. This pathway begins with the phosphorylation of JAK1 and TYK2 (non-receptor tyrosine-protein kinase) on the cytoplasmic domains of the heterodimeric receptor subunits. This is followed by the phosphorylation and dimerization of STAT1/2. In the type III IFN signaling pathway, activated STAT1 and STAT2 recruit IRF-9 to form a complex called the Interferon-Stimulated Gene Factor 3 (ISGF3). ISGF3 translocates to the nucleus and binds to the interferon-sensitive response element (ISRE) in the promoters of interferon-stimulated genes (ISGs). This results in gene transcription and translation into host proteins with antiviral effector activity, including ZAP, TRIMs, 2’-5’-Oligoadenylate Synthetase 1 (oAS1), and ribonuclease L (RNaseL). These proteins help control viral replication and dissemination for early immune defense. Some ISGs are already elevated at basal levels or can be modulated by IRF3 [92,93,94]. It is known that IRF3 binds to ISRE in the human ZAP promoter during viral infection [11], and type I IFN has a greater effect on inducing ZAP-S expression compared to ZAP-L, possibly through transcription regulation, alternative splicing, or polyadenylation [16,17,40,95] (Figure 6).

Schwerk et al. (2019) [17] found that the two isoforms of the ZAP protein have distinct functions, which is due to the absence of the C-terminal prenylation motif in ZAP-S. The presence of the PARP-like domain in ZAP-L contains a cysteine (CaaX) motif, which mediates S-farnesylation and targets it to endolysosome membranes or the endoplasmic reticulum [17,18,25,42]. ZAP, particularly ZAP-S, is also stimulated by dsRNA and dsDNA in HEK293T and plays a critical role in amplifying RIG-I activity, as discussed in the next section [40]. Furthermore, it was found that when cells were stimulated with IFN and nucleic acids, the expression of ZAP-S was upregulated [31,40,46]. However, ZAP-S is less effective in limiting murine leukemia viruses (MLVs) and alphaviruses compared to ZAP-L, demonstrating that the isoforms inhibit infections and respond to IFNs in different ways [15,96]. Despite the different antiviral activities of the ZAP isoforms, depending on the virus, there is no difference between the isoforms in terms of the intensity of stimulating the expression of type I IFN [16]. In BHK cells (Baby Hamster Kidney fibroblasts), IFN-I stimulation is necessary for ZAP activity, whereas in Rat2 and HEK293 cells, it is already functional [13,38,62]. Therefore, one could argue that specific cellular factors may interfere with ZAP action.

### 7.2. Retinoic Acid-Inducible Gene I (RIG-I)

RIG-I is part of the DEx(D/H) box helicases family. It consists of two N-terminal CARD domains, followed by a central RNA helicase domain and a C-terminal Repressor domain (RD) with ATPase activity that recognizes 5’-triphosphorylated RNA. TRIM25 [97] and K6-linked Ub chains polyubiquitinate the second CARD (CARD2), resulting in RIG-I oligomerization. In its activated state, RIG-I recruits and binds to the mitochondrial antiviral-signaling protein (MAVS) through its CARD domain (caspase activation and recruitment domain) to the mitochondrial antiviral-signaling protein (MAVS), leading to the activation of TBK1-IKKϵ and IKKα-IKKβ complexes. These complexes activate IRF-3/IRF-7 and NF-κB, respectively. Translocated IRF-3 and IRF-7 stimulate the synthesis of type I IFNs, which bind to their respective receptors and activate intracellular signaling, leading to the transcription of ISGs. The products of these ISGs have antiviral activity by reducing viral spread [98] (Figure 6).

Riplet also has an activity in regulating RIG-I signaling by acting as an E3 ubiquitin ligase. It leads to K63-linked polyubiquitination of RIG-I RD [99]. Furthermore, according to Oshiumi et al. (2013) [99], Riplet is a condition for TRIM25 to activate RIG-I signaling [99]. Cadena et al. (2019) [100] demonstrated that Riplet, as an E3 ligase, acts as a co-receptor that collaborates in the oligomerization of RIG-I, amplifying antiviral signaling. They further demonstrated that TRIM25 was not essential for full-length RIG-I signaling. Instead, ectopic expression of TRIM25 acts as a moderate stimulator of signaling mediated by fragments of RIG-I CARD [100].

Riplet has also been suggested to act as a cofactor of ZAP [101]. Buckmaster and Goff (2022) [101] demonstrated that overexpression of Riplet increased the antiviral activity of ZAP in human cells infected with VSVG-pseudotyped HIV-luc reporter virus [101]. The mechanisms by which Riplet enhances ZAP’s antiviral activity need further investigation. There is no relationship between Riplet’s E3 ubiquitin ligase function and ZAP’s ability to inhibit the virus [101]. However, Riplet binds to ZAP through its C-terminal P/SPRY domain, and this interaction is important for the inhibition of HIV-1 reporter virus [101]. Riplet also interacts with TRIM25, and both enhance ZAP-mediated inhibition of HIV-1-luc reporter virus. Further investigation is needed to determine if a complex formation between TRIM25, Riplet, and ZAP activates an antiviral state.

ZAP-S increases IFN production in human HEK293T cells in the presence of 5’-triphosphate RNA, which directly binds to RIG-I [40,102]. This is likely due to the interaction between the N-terminal domain of ZAP-S and both the helicase domain and the C-terminal region of RIG-I, promoting oligomerization and ATPase activity and, thereby, enhancing the downstream signaling pathway [40] (Figure 6).

ZAP-S stimulates the type I interferon response mediated by RIG-I in human primary CD14^+^ monocytes and fibroblasts [40]. ZAP-S has dual actions: cleavage of vRNA with the aid of p72 RNA helicase and activation of the antiviral innate immunity pathway by binding to RIG-I [44,81]. However, ZAP-S’s involvement in RIG-I activation appears to be dependent on the cell type and species. Lee et al. (2013) [103] found that in primary mouse cells, ZAP-S did not affect the type I IFN response mediated by RIG-I, since in mouse cells lacking ZAP, when infected with viruses recognized by RIG-I, IFN-β and CXCL10 were produced normally [103].

The promoter region of the gene encoding ZAP-S contains interferon-stimulated response elements (ISREs) and interferon regulatory factor (IRF)-binding elements. Further studies are needed to understand the regulation of ZAP-S [40]. ZAP also facilitates greater binding of RIG-I to dsRNA and recruits TRIM25 for ubiquitination and subsequent activation of RIG-I [104]. More studies are needed to investigate why ZAP-L does not regulate RIG-I in the same manner.

### 7.3. OAS1–Rnasel Antiviral Pathway

2’, 5’-Oligoadenylate synthetase 1 (OAS1) is an ISG that is expressed at low levels and is upregulated by type I and type III IFNs. It is found in the cytosol as a monomer in its inactive form. Upon activation by dsRNA, it undergoes oligomerization into a tetramer and uses ATP to synthesize 2’, 5’-oligoadenylate molecules. These molecules then bind to the inactive and monomeric form of RNaseL, which undergoes dimerization and is allosterically activated. RNaseL, which is present in the cytoplasm, is constitutively expressed and, when activated, degrades a wider range of viral (ssRNA) and cellular RNAs, thereby limiting viral replication [105]. Odon et al. (2019) [27] demonstrated that RNaseL mediates the decrease in E7 virus with high CpG and UpA content. They also identified that ZAP binds to the mutated virus and inhibits it. This decrease in viral replication was dependent on the expression of ZAP and RNaseL in vitro [27]. Additionally, they found that in the absence of ZAP in cells, there was a greater increase in constitutive expression of RNaseL, suggesting that these pathways may work together to attenuate viruses with enriched dinucleotide frequency [27]. It is important to note that some viruses can neutralize RNaseL activation [106,107], which allows ZAP to serve as an alternative pathway in innate immunity for controlling viral infections.

## 8. Antiviral Activity of ZAP in Different Virus Families

In the following section, we will discuss what is known so far about the inhibition of viruses by ZAP and associated cofactors. The list of these viruses and ZAP function during the infection can be found in Table 1.

### 8.1. Retroviridae Family

#### 8.1.1. Moloney and Murine Leukemia Virus (MoMLV or MuLV or MLV)

This virus is the cause of lymphoid leukemia in mice. A genetic screen for host factors with antiviral activities identified the ZAP protein in an overexpression of rat cDNA. This overexpression decreased Moloney murine leukemia virus (MuLV) replication, suggesting that the ZAP protein acts in the post-transcriptional viral mRNA step in the cytoplasm. No effect of ZAP on viral RNAs in the nucleus was observed, indicating that ZAP does not interfere with the initial transcript. Rat2 cells overexpressing rNZAP-Zeo, when infected with MLV, showed a decrease in the amount of virus mRNA in the cytoplasm. ZRE was mapped to the 3’-LTR (long terminal repeat) in MLV [44]. Human ZAP-L exhibited stronger antiviral activity than ZAP-S against MLV LTR-driven luciferase expression. Lee et al. (2013) [103] found that ZAP detects MLV transcripts and directs them to the exosome. ZAP was also found to be located in the RNA granules. The N-terminal domain of ZAP was identified as responsible for targeting MLV transcripts to RNA granules, along with the exosome (in vitro) components, and this activity was independent of RIG-I [103]. Given the viral targeting of RNA granules, it is important to evaluate how the different ZAP isoforms act against MLV infection in vitro and in vivo. This study highlights the significance of RNA granules as an important cytoplasmic antiviral hub.

#### 8.1.2. Human Immunodeficiency Virus Type 1 (HIV-1)

This virus is responsible for causing AIDS, a serious disease that lowers the immune system, making the individual more susceptible to opportunistic infections. HIV-1 has a low frequency of CpG dinucleotides [124]. Antzin-Anduetza et al. (2017) [112] demonstrated that increasing CpG dinucleotides after codon modification in HIV-1 led to a decrease in infectivity and virus replication in HeLa cells [112]. They hypothesized the presence of a sensor that detects the CpG dinucleotide sequence in the viral RNA, which could explain the results found. Takata et al. (2017) [22] observed the same results as the other mentioned authors after generating a new version of the genomic RNA of HIV-1 mutant (CG-enriched segment in mutant L). They found that the virus containing large amounts of CG dinucleotides, compared to the wild virus, experienced depletion of cytoplasmic unspliced viral RNA and impairment in viral RNA replication. When they inhibited the expression of the ZAP protein in the host cell, there was replication of those viruses with increased CG dinucleotides [22]. The amount of CG dinucleotides in the HIV-1 genome is generally low, but it is high in the 5’UTR region. This may explain the disagreement found in the literature regarding whether the ZAP protein inhibits HIV-1 replication under normal conditions [22,55]. Additionally, it is important to observe the viral titer used in infections in the studies because, depending on the titer, ZAP protein activity may be depleted, and its action on the inhibition of viral replication may not be observed. Further in vivo studies on the role of ZAP in inhibiting HIV-1 viral replication are important to verify a possible effective target against HIV-1.

Ficarelli et al. (2020) [125] found that when CpG dinucleotides were in the 5’ region of HIV-1 env, the virus underwent an antiviral action by ZAP more efficiently than when CpGs were inserted into other regions of the viral genome, although high levels of ZAP can bind to other regions containing CpG in the viral genome. This also demonstrated that the number of CpGs was not related to greater antiviral activity by ZAP, although it is not known if there is a specific number of CpGs for ZAP to bind and induce viral RNA degradation [125]. However, the insertion of CpG dinucleotides can generate live attenuated vaccines. Kmiec et al. (2020) [52] found that the amount of CpG in the region of approximately 700 bases at the 5’ end of the env gene of the HIV-1 strain determines the sensitivity of this strain to the ZAP protein. The increase in the amount of CpG dinucleotides in this same region was related to a reduction in the expression of ENV mRNA, as well as the production of virus proteins (p24 and Env). Furthermore, HIV-1 was inhibited by ZAP in human cells. Thus, the inclusion of these dinucleotides in this region of the HIV-1 ENV may negatively affect disease progression, favoring the host. More studies are needed to validate this hypothesis [52]. Furthermore, the mechanisms of how the ZAP protein inhibits these modified viruses need to be investigated.

According to Zhu et al. (2011) [55], both ZAP isoforms limit vesicular stomatitis virus G protein (VSV-G)-pseudotyped HIV-1 vector NL4-3-luc infection through RNA degradation mediated by the recruitment of PARN deadenylase, exosome, and decapping complex through the p72 helicase [55]. Sertkaya et al. (2021) [126] found that endogenous ZAP efficiently inhibited CpG-high HIV-1 in human cells and that the overexpression of ZAP did not inhibit lentiviral vector titer [126]. Kmiec et al. (2021) [25] demonstrated that ZAP-L, due to its PARP domain and CaaX, directs this isoform to vesicular structures, regulates the binding with TRIM25 and KHNYN, and is important for the antiviral activity of CpG-enriched HIV-1 [25].

#### 8.1.3. Avian Leukosis Virus Subgroup J (ALV-J)

This virus can have a significant impact on the poultry industry and cause extensive damage to the economy. It has been discovered that ZAP inhibits the replication of ALV-J in vitro. Zhu et al. (2020) [113] also found the same result in vivo, where overexpressing ZAP made animals resistant to ALV-J infection. Additionally, ALV-J induced the production of ZAP in lymphocytes, and overexpressing ZAP helped with the proliferation of T lymphocytes but not B lymphocytes during ALV-J infection. However, ZAP promoted the production of antiviral antibodies and stimulated the secretion of interleukins IL-2, IL-4, and IL-21 mediated by activated T lymphocytes, suggesting that ZAP also has an immunomodulatory function. Zhu et al. (2022) [127] discovered that ZAP improves T-cell immunosuppression caused by ALV-J and activates these cells through the norbin-like protein (NLP)-protein kinase C delta (PKC-δ)-nuclear factor of activated T cell (NFAT) pathway [127].

#### 8.1.4. Human T-lymphotropic Virus Type 1 (HTLV-1)

HTLV-1 was the first retrovirus discovered to cause cancer, specifically adult T-cell leukemia/lymphoma, and it also causes damage to the nervous system. Miyazato et al. (2019) [114] found that overexpressing ZAP in JEX22 cells resulted in reduced synthesis of the HTLV-1 p19 protein. Knocking down ZAP using two different siRNAs improved p19 production and increased virus production in the culture supernatant, suggesting that ZAP acts as a defense mechanism in host cells.

### 8.2. Flaviviridae Family

#### 8.2.1. Japanese Encephalitis Virus (JEV)

JEV is a virus that can be transmitted through mosquito bites and is more common in rural and agricultural areas. It can cause brain swelling and is one of the viruses that can lead to encephalitis in humans. Chiu et al. (2018) [10] demonstrated that overexpressing ZAP in certain human cell lines inhibited JEV infection. They also identified the 3’-UTR of the JEV genome as the ZAP-responsive element (ZRE). ZAP affected JEV translation, interfered with vRNA stability, directed vRNA to the 3’-5’ RNA exosome-mediated degradation pathway, and stimulated the synthesis of IFN-β, TNF-α, and IL-6, which may contribute to the host’s response to JEV infection. This was the first study to identify ZAP-sensitive flavivirus [10]. Yang et al. (2022) [9] found that double mutations (abbreviated as KY) within the ZnF2 of ZAP-S and ZAP-L increased JEV translation [9]. Okudera et al. (2022) [43] discovered that when siRNA against ZAP was used in brain microvascular endothelial cells, there was an increase in JEV titers, suggesting that ZAP may contribute to the innate immune response against JEV by preventing the virus from entering the brain [43]. However, the mechanisms by which ZAP inhibits virus propagation still need to be elucidated in vitro and confirmed in vivo.

#### 8.2.2. Zika Virus (ZIKV)

In some cases, Zika can cause symptoms such as a low-grade fever, rash, headache, and joint pain. In other cases, it can lead to paralysis, specifically Guillain-Barré syndrome. In pregnant women, Zika can result in birth defects. Trus et al. (2020) [115] observed that infection was weakened in RD cells when modified ZIKV with a higher frequency of CpG dinucleotides was used. The ZIKV genomic regions encoding E and NS1 proteins were recoded to increase the number of CpG dinucleotides. Additionally, in vivo experiments showed that the infection was more attenuated in adult mice compared to young mice, and the expression of ZAP protein in the brain depended on the age of the animal. This needs further evaluation in other tissues. The use of these modified viruses resulted in longer survival of the animals, but they also triggered a significant innate and adaptive immune response.

As discussed in another section, further studies are necessary to assess the safety of using CpG-recoded virus vaccine candidates, as this may have a significant impact on vaccine effectiveness [115,128]. Fros et al. (2021) [26] found that viral replication was restored in vertebrate cells lacking ZAP (A549 ZAP knockout cells) compared to vertebrate cells with ZAP (A549 cells), where viral replication decreased. When the kinetics of viral replication (WT ZIKV) were examined in mosquito cells, viral titers increased. In in vivo experiments, IFNAR^−/−^ mice (deficient in the interferon-α/β receptor) infected with ZIKV mutants with a high frequency of CpG dinucleotides showed faster weight recovery and a lower peak of viremia compared to animals infected with WT ZIKV. When these animals infected with ZIKV mutants were subsequently infected with WT ZIKV, there was no change in testicular size, suggesting that the modified ZIKV (ZIKV mutants) could be used as live attenuated vaccines [26].

### 8.3. Togaviridae Family

#### 8.3.1. Sindbis Virus (SINV)

SINV is transmitted by mosquitoes, specifically Culex and Culiseta species, and is related to Pogosta disease, Ockelbo disease, and Karelian fever. This virus causes symptoms such as arthralgia, skin rash, and malaise. Bick et al. (2003) [7] discovered that cells expressing the amino-terminal portion of ZAP fused to the zeocin resistance gene product (NZAP-Zeo) inhibited replication of Sindbis virus (SIN), Semliki Forest virus (SFV), and Ross River virus (RRV), regardless of the MOI used in the infection. This inhibition was observed compared to control cells (Rat2-HA-Zeo cells expressing the vector alone). Additionally, replication of Venezuelan Equine Encephalitis (VEE) was inhibited in cells that expressed NZAP-Zeo. The expression of structural genes of the alphavirus was not found to be essential for the inhibitory activity of the ZAP protein [7].

MacDonald et al. (2007) [38] found that ZAP overexpression did not affect virion production in BHK cells deficient in IFN synthesis. This indicates the significance of type I IFN signaling in viral control. The presence of both ZAP and IFN had a greater efficiency in controlling SINV in IFN-α pretreatment, suggesting the involvement of an unknown ISG. Furthermore, the ZAP protein in BHK cells was able to prevent viral translation. IFN-α signaling was also found to be important for enhancing the antiviral activity of ZAP in Stat1-deficient MEF cells. In these cells, ZAP overexpression prevented virion production even in the absence of IFN, unlike in BHK cells. These results emphasize the importance of assessing the antiviral activity of ZAP in conjunction with other factors, such as IFN signaling and ZAP cofactors, in different cell lineages to gain a comprehensive understanding of the role of ZAP.

Based on previous findings by Zhang et al. (2007) [37] that IFN-α/β repressed Sindbis virus (strain TR339) replication and inhibited viral translation protein kinase R (PKR) independently, the authors investigated other host factors that could possess antiviral activity and be upregulated by the IFN pathway. They discovered that the ZAP protein prevented SINV replication in vitro and provided protection against infection in mice [37]. Therefore, Zhang et al. (2007) [37] found that ZAP was upregulated in dendritic cells derived from mouse bone marrow infected with SINV or treated with IFN, but it was undetectable without these conditions [37]. Kozaki et al. (2015) [129] also demonstrated in their study that ZAP was an important antiviral protein against SINV. It inhibited viral replication in primary MEF cells, and the N-terminal domain of ZAP played a crucial role in this control. Similar to the findings of MacDonald et al. (2007) [38], ZAP exerted this function independently of IFN type I signaling, reducing SINV RNA levels. In vivo, animals lacking ZAP were found to be more susceptible to infection [129]. Wang et al. (2016) [109] showed that SINV replicated in lymphoid tissues but not in the brain. This restriction may be attributed to increased levels of type I IFNs induced by the infection [109].

During cellular stress, both ZAP isoforms were identified in cytoplasmic stress granules, which may be functionally important for their antiviral activity [42,47,130]. ZAP not located in these stress granules did not inhibit SINV replication [130]. This finding justifies the observations made by Charron et al. (2013) [18] and others, who identified that human ZAP-L exhibited stronger antiviral action against SINV infection in MEF cells compared to ZAP-S [15,16,18]. This result aligns with the findings of Li et al. (2019) [16] but in relation to viral translation. In the early stages, ZAP-L was more effective in preventing the translation of SINV in 293T cells [31]. This difference may be due to ZAP-L’s PARP domain with the S-farnesylation motif, which directs it to the endolysome membranes [18]. In contrast, ZAP-S, which is normally not prenylated, does not possess this motif. When using ZAP knockout mice, Kozaki et al. (2015) [129] observed an increase in SINV replication after infection [129]. Li et al. (2017) [20] demonstrated that both ZAP isoforms interact with TRIM25 through their SPRY domain, and this interaction is crucial for their antiviral activity. It enhances their ability to inhibit the translation of the incoming SINV genome [20].

#### 8.3.2. Semliki Forest Virus (SFV)

This virus is transmitted in humans through mosquito bites and causes mild symptoms in humans. Human ZAP- L restricts SFV (SFV was made using the DNA-based Semliki Forest Virus vectors (pSMARTlacZ and pSCAHelper)) infection in HeLa cells more efficiently than ZAP-S, possibly due to the presence of the PARP domain, requiring further studies that seek the mechanism for such a proposal [15]. With that, and given the scenario of alphavirus epidemics, ZAP-L can be thought of as a target for therapeutic intervention involving alphavirus-induced diseases.

### 8.4. Filoviridae Family

#### Ebola Virus (EBOV) and Marburg Virus (MARV)

Ebola is a severe disease caused by the Ebola virus, resulting in organ failure, heavy bleeding, and, ultimately, fatality. Similarly, the Marburg virus is the cause of Marburg hemorrhagic fever and shares similarities with Ebola in terms of symptoms [6]. In a study conducted by Muller et al. (2007) [6], it was demonstrated that ZAP, when expressed in 293TRex cells with full-length ZAP, inhibited the replication of both EBOV and MARV. This inhibition was achieved by reducing the mRNA levels of both viruses. The study also highlighted the importance of intact motifs ZnF2 and ZnF4 for the efficient antiviral activity of ZAP. Apart from the two isoforms of ZAP mentioned in this review, Li et al. (2019) [16] discovered two additional isoforms in humans: ZAP-XL (extralong) and ZAP-M (medium). When evaluating the antiviral activity of ZAP against the Ebola virus, specifically in terms of inhibiting viral transcription and replication, all isoforms exhibited similar effects [16]. In a study by Galão et al. (2022) [12], it was demonstrated that TRIM25 induces the dissociation of the viral nucleoprotein (NP) from the Ebola virus RNA. This dissociation exposes the viral genome, enabling the binding of ZAP to the CpG dinucleotide, ultimately leading to the inhibition of viral replication. The authors also found that ZAP-L restricts EBOV more effectively than ZAP-S [12].

More studies, primarily in vivo, are necessary to comprehend the ZAP protein as an antiviral host factor for the development of ZAP-based therapeutics.

### 8.5. Picornaviridae Family

#### 8.5.1. Coxsackievirus B3

This virus belongs to the enterovirus family and is capable of infecting various parts of the body, including the skin, nails, eyes, airways, heart, and throat. Transmission occurs through person-to-person contact, primarily due to poor hand hygiene and contact with feces-contaminated surfaces. Infection with this virus can result in the development of conjunctivitis, meningitis, and myocarditis. Li et al. (2015) [14] discovered that infection with this virus triggers the expression of ZAP, both in vivo and in vitro. Interestingly, they also observed that animals with an overexpression of ZAP demonstrated increased resistance to viral replication and myocarditis. In vitro experiments revealed that ZAP inhibits viral replication, with the regions in CVB3 RNA that interact with ZAP identified in both the 3’ UTR and 5’ UTR [14]. However, further studies are necessary to confirm these findings and explore the involvement of ZAP protein cofactors and other innate immunity pathways. Consequently, ZAP has the potential to be a target for the development of therapies for viral myocarditis.

#### 8.5.2. Echovirus 7 (E7)

This virus can cause fever, skin rash, loss of appetite, and other symptoms such as acute upper respiratory tract infection and enteritis. Atkinson et al. (2014) [89] observed that increasing the frequencies of CpG dinucleotides in the coding regions of the E7 virus resulted in a decrease in virus replication in A549 cells. This demonstrates that although this enterovirus can suppress the cell’s immune system, it was unable to avoid recognition and experienced reduced replication when rich in CpG dinucleotide sequences [89]. In vivo studies are necessary to confirm whether modifying these viruses can limit their spread. In Tulloch et al. (2014) [116], echovirus 7 replication was affected when the virus was modified to have increased frequencies of CpG and UpA dinucleotides [116]. Assessing the innate immune response to these virus modifications is crucial for developing safely attenuated live vaccines.

#### 8.5.3. Enterovirus A71 (EV-A71)

The main symptoms generated by EV-A71 infection are wounds in the body, and it generates a disease called “hand-foot-mouth disease”, causing damage to the gastrointestinal system and causing stomatitis. It was demonstrated that in RD and HeLa cells, when infected with EV-A71, it increased the levels of ZAP-L mRNA. However, the 3C protease (3Cpro) of the virus led to cleavage at position Gln-369 of both ZAP isoforms, thereby inhibiting the antiviral activity of ZAP [117].

#### 8.5.4. Poliovirus

Poliovirus is the agent that causes poliomyelitis in humans, affecting the nervous system, and can lead to muscle weakness or paralysis. It is transmitted by direct contact with feces or secretions eliminated through the mouth. Burns et al. (2009) [118] found that when modifying the laboratory reference strain of wild poliovirus type 2 (MEF-1) with increasing frequencies of CpG dinucleotides, there was a decreased replicative fitness in HeLa cells, as well as a reduction in poliovirus infectivity. Therefore, this genetic modification can be useful for the production of attenuated vaccines [118].

### 8.6. Orthomyxoviridae Family

#### Influenza A Virus

This virus causes influenza in humans, which is an acute respiratory disease. Symptoms include fever, cough, sore throat, body aches, and malaise. In some cases, complications can arise, leading to hospitalization and death. In a study by Liu et al. (2015) [110], it was discovered that the C-terminal domain of ZAP-L provides moderate antiviral activity against the influenza virus. This occurs through the interaction between the C-terminal portion of ZAP and the proteins PB2 (polymerase basic protein 2) and PA (acidic protein polymerase) of the influenza virus polymerase complex, resulting in the degradation of these proteins. Additionally, it was found that in the absence of the C-terminal portion of ZAP, the virus replicated in cell culture. However, further research is necessary to confirm whether this replication was caused by the degradation of PB2 and PA proteins of the virus, as the PB1 protein of the virus inhibited the activity of ZAP-L [110].

Other proteins are also involved in neutralizing the antiviral activity of ZAP, such as the NS1 protein of the influenza A virus. The NS1 protein inhibits the interaction between ZAP-S and the target viral RNA, which may explain why downregulated ZAP-S has little effect on virus replication in influenza A [131]. Additionally, the RTA protein from the MHV-68 virus [132] and the 3C protein from enterovirus (EV)-71 [117] are also involved in this process, as discussed in this review, along with other proteins. The study of these interactions between ZAP and viral proteins is necessary for the study of antiviral therapy.

In a study by Gaunt et al. (2016) [133], a decrease in IAV enriched with CpG and UpA dinucleotides was observed in vitro. They also found that when mice were infected with IAV modified to have an increased content of CpG dinucleotides, the pathogenicity of the virus decreased, as well as the viral load in lung tissue. However, it did cause an adaptive immune response similar to that of the wild virus [133]. Similar results were found in the work of Sharp et al. (2023) [111]. They discovered that the enrichment of CpG dinucleotides in segment 1 of the influenza A virus (IAV) genome (CpGH IAV) led to a defect in ZAP-S-dependent viral replication in human cells, but it did not affect IAV virion assembly. They also found that the anti-CpG activity of the ZAP protein was independent of the type I IFN pathway. In vivo, animals showed mild or absent clinical signs after being infected with the CpGH IAV [111]. These results support the continuation of research aimed at the development of live attenuated vaccines.

### 8.7. Poxviridae Family

#### Vaccinia Virus Ankara (MVA)

MVA is an attenuated vaccine of a poxvirus. In A549 cells, ZAP limits MVA since ZAP interferes with the construction of infectious MVA virions when C16 protein is not present, as the protein antagonizes ZAP [119].

### 8.8. Hepadnaviridae Family

#### Hepatitis B Virus

Hepatitis B is a disease caused by the hepatitis B virus (HBV), and it affects the liver. It is also considered a sexually transmitted infection, as HBV is present in blood, secretions, and bodily fluids. In chronic cases, it can lead to liver failure and cancer. According to Mao et al. (2013) [31], in vitro studies have shown that overexpression of ZAP can control viral replication in HepG2 cells by reducing viral RNA through post-transcriptional mechanisms. They also found that the N-terminal domain is important for this antiviral activity [31]. To evaluate the role of ZAP against HBV in vivo, Chen et al. (2015) [120] demonstrated that ZAP protein decreased HBV DNA replication intermediates in mouse liver tissue [120]. Further studies are needed to evaluate the presence of ZAP cofactors for its antiviral activity, as the HBV ZRE is located near both ends of pgRNA. Since human ZAP isoforms have shown differences in antiviral activity against certain viruses, Li et al. (2019) [16] identified that ZAP-L and ZAP-XL have a greater efficiency as antiviral agents against HBV compared to ZAP-S and ZAP-M [16]. More in vivo studies are required to understand the antiviral mechanisms of ZAP and the cofactors involved. This will help determine whether ZAP can be a therapeutic target for patients with viral hepatitis B.

### 8.9. Herpesviridae Family

#### Murine Gammaherpesvirus 68

MHV-68 is a natural pathogen of murid rodents. Its life cycle includes latent and lytic phases. It has been observed that ZAP binds to M2 mRNA, which is expressed during the latency phase of MHV-68, and this binding leads to a decrease in M2 expression. This suggests that ZAP plays a role in modulating the latency of MHV-68. However, there may be other regulatory mechanisms at play, and further studies are needed to investigate this [33]. In a study by Xuan et al. (2013) [132], it was found that infection of HEK293T cells by MHV-68 stimulated the expression of ZAP. However, RTA, a replication and transcription activator produced by MHV-68 during the early stages of the lytic cycle, negatively interferes with the self-interaction of the N-domain of ZAP. This is significant because ZAP needs to be in a dimer shape formed by its N-terminal tails in order to exert its antiviral activity [48,57]. This may explain why the authors of the study did not observe inhibition of MHV-68 replication by ZAP. Nevertheless, further research is required to fully understand the mechanisms behind this inhibition of ZAP’s antiviral activity, which is induced by RTA.

### 8.10. Coronaviridae Family

#### Severe Acute Respiratory Syndrome Coronavirus 2

COVID-19 is a disease caused by infection with severe acute respiratory syndrome coronavirus 2 (SARS-CoV-2). It can lead to mild to severe symptoms and even death. ZAP, a protein, has been found to recognize viral RNA with a high frequency of CpG dinucleotides. Nchioua et al. (2020) [51] discovered that ZAP and its cofactors KHNYN and TRIM25 are expressed in Calu-3 cells infected with SARS-CoV-2. They also found that the virus increased the expression of ZAP-S, and this increase was greater when treated with IFN-γ and IFN-β. When ZAP was knocked down, there was an increase in viral RNA, and ZAP’s restriction of the virus was more effective in the presence of type II IFN. Additionally, endogenous ZAP-L was more effective at restricting the virus than ZAP-S in HEK293T cells, which supports the findings of Kmiec et al. (2021) [25]. Furthermore, ZAP-L requires the CaaX box to inhibit SARS-CoV-2 [25]. The authors suggest that ZAP restricts this virus because SARS-CoV-2 has a high amount of CG dinucleotides in the 3’ end region. Zhang et al. (2021) [134] observed that Huh7 cells overexpressing ZAP-L and co-transfected with replicon RNA and N protein mRNA showed reduced replication of SARS-CoV-2 replicon in these cells [134] (Zhang et al., 2021). Zheng et al. (2021) [135] demonstrated that ZAP can interact with the N gene of SARS-CoV-2, which contains a higher frequency of CpG than the average seen in the SARS-CoV-2 genome [135]. Kamel et al. (2021) [136] showed that cells infected with SARS-CoV-2 had an upregulation of ZAP, suggesting the involvement of this protein in the antiviral response [136].

In contrast to other authors, Lee et al. (2021) [122] and Zimmer et al. (2021) [24] found that ZAP-S is more effective at reducing SARS-CoV-2 replication than ZAP-L. However, the action of the TRIM25 cofactor is required for this effect, and further research is needed to understand the mechanism by which this occurs [122]. Additionally, Zimmer et al. (2021) [24] demonstrated that ZAP-S directly regulates translational frameshifting in cells infected with SARS-CoV-2, thereby inhibiting the virus [24].

The molecular mechanisms underlying the restriction of SARS-CoV-2 by ZAP still need to be elucidated, as well as the different antiviral responses observed between ZAP isoforms and the involvement of ZAP cofactors in viral restriction [51]. Afrasiabi et al. (2022) [137] showed that the low frequency of CpG dinucleotides in SARS-CoV-2 is not a result of evolutionary pressure mediated by ZAP activity. This leaves the question unanswered and calls for further investigation into other evolutionary forces that may have led to the decrease in CpG frequency in SARS-CoV-2 [137,138].

Based on these findings, there is potential for therapeutic intervention through genomic manipulation.

### 8.11. Paramyxoviridae Family

#### 8.11.1. Sendai Virus

This virus naturally infects the respiratory tract of rodents and has been studied to serve as an experimental intranasal vaccine. Li et al. (2019) [16] identified that ZAP isoforms in humans do not differ in stimulating the expression of IFN type I in cells infected with Sendai virus (SeV) [16]. Wang et al. (2010) [11] also found that hZAP mRNA was upregulated by SeV infection in vitro and appeared to be dependent on IRF3 [11].

#### 8.11.2. Newcastle Disease Virus (NDV)

NDV is transmissible to humans and can cause flu-like symptoms. Hayakawa et al. (2011) [40] found that ZAP-S binds to RIG-I in HEK293T cells infected with NDV and that in a situation of downregulated expression of ZAP-S, these cells infected with NDV showed a decrease in RIG-I oligomers. This demonstrates that ZAP-S is important in the oligomerization of RIG-I, followed by downstream activation of RIG-I. In ZC3HAV1-knockout cells, they found that NDV infection decreased the expression of IFN-β, TNF-α, and CXCL10 [40].

## 9. Challenges and Perspectives

In this review, we demonstrate that human ZAP restricts the expression and replication of many viruses. This restriction is related to the sequence of CpG dinucleotides and the integrity of the structure that accommodates that sequence. Some viruses have the ability to encode proteins that oppose ZAP, while others mask the region in the viral RNA recognized by ZAP. We discuss the differences between ZAP isoforms in terms of their antiviral activity and intracellular location, which directly affects their antiviral function. The presence of cofactors and associated immune pathways also plays a role in enhancing ZAP’s efficiency against viruses. However, previous studies have mainly evaluated the antiviral activity of ZAP-S and ZAP-L individually, which may obscure interactions between the isoforms in cells that express endogenous ZAP.

It is also important to investigate whether the cofactor requirements of ZAP are dependent on cell type. Therefore, it is necessary to evaluate other techniques that allow the assessment of individual isoforms in the absence of endogenous ZAP protein expression. Additionally, using overexpression of ZAP in assays may result in non-physiological levels. Moreover, in vitro studies have shown that the requirement of other immune pathways and cofactors for ZAP activity varies depending on the cell line used, making it challenging to compare studies and elucidate the antiviral mechanism of ZAP. Different cells may have other factors that regulate the antiviral activity of ZAP.

Finally, while many works have evaluated the activity of ZAP in vitro, it is crucial to assess its activity in vivo. This is because there are proteins that can regulate the activity of ZAP, as well as the involvement of cofactors and immune pathways. It is important to understand how these factors are related in different viral contexts and within more complex biological systems. Determining the antiviral mechanism of ZAP and its responses can provide therapeutic targets.

As discussed in the text, recoding virus genomes by altering the frequency of CpG dinucleotides can increase their susceptibility to the antiviral role of the ZAP protein. This can lead to a reduction in viral replication while triggering an acquired immune response in the host. Therefore, creating CpG-recoded viruses can be a valuable tool in developing live attenuated virus vaccine candidates. Understanding the RNA sequences that serve as ZAP binding points is important for introducing other ZREs into the recoded virus. Overall, a deeper understanding of the antiviral role of the ZAP protein through experimental and clinical studies will enable its use in the context of human health.

## Figures and Tables

**Table 1:** Function of ZAP during a viral infection.

Virus Family		Effect of ZAP on Viruses	Cofactors Required by ZAP	Associated Immune Pathways	References
***Togaviridae***	*Sindbis virus (SINV)*	- Inhibits SINV replication and translation; - Stronger activity of ZAP-L compared to ZAP-S.	TRIM25	Type I IFN	[7,15,16,18,20,38,108,109]
	*Semliki Forest virus (SFV)*	- ZAP-L restricts SFV infection more efficiently than ZAP-S in HeLa cells.	Not measured	Not measured	[7,15]
	*Ross River virus (RRV)*	- RRV showed a consistent 2- to 3-log-unit reduction in titer in ZAP-expressing cells compared to its titer in control cells.	Not measured	Not measured	[7]
*** Orthomyxoviridae ***	*Influenza A virus*	- C-terminal domain of ZAP-L provides moderate antiviral activity; - Decrease in IAV enriched with CpG and UpA dinucleotides in vitro; - siRNA-mediated knockdown of ZAP-S expression impaired the induction of - IFN-β and IFN-α1 mRNA and IFN-β protein in response to infection with influenza virus; - CpG dinucleotides in segment 1 of the influenza A virus (IAV) genome (CpGH IAV) led to a defect in ZAP-S-dependent viral replication in human cells; - In vivo, animals showed mild or absent clinical signs after being infected with the CpGH IAV.	Not measured	- RIG-I-mediated type I interferon response	[40,103,110,111]
***Retroviridae***	*Moloney and murine leukemia virus (MMLV or MuLV)*	- Diminishes vRNA in the cytoplasm; - ZAP-L has stronger antiviral activity than ZAP-S; - Inhibits replication, = acts in the post-transcriptional viral mRNA step in the cytoplasm.	- Exosome	- Independent of RIG-I.	[13,15,30,103]
	*Human immunodeficiency virus type 1 (HIV-1)*	- Decrease in infectivity and virus replication of high CpG-HIV-1; - The number of CpG within a region of ∼700 bases at the 5′ end of the env gene determines ZAP sensitivity of HIV-1 strains; - PARP and CaaX box were essential for antiviral activity against CpG-enriched HIV-1; - Both ZAP isoforms limit vesicular stomatitis virus G protein (VSV-G)-pseudotyped HIV-1 vector NL4-3-luc infection.	- PARN deadenylase, - Exosome; - p72 helicase; - E3 ubiquitin ligase TRIM25; - KHNYN.	Not measured	[22,25,52,55,112]
	*Avian leukosis virus subgroup J (ALV-J)*	- Overexpression of ZAP inhibited ALV-J replication and reduced the associated inflammatory damage in vivo; - ZAP activated cytokine secretion by T lymphocytes; - ZAP indirectly promoted anti-ALV-J antibody generation.	Not measured	Not measured	[113]
	*Human T-lymphotropic virus type 1 (HTLV-1)*	- A dose-dependent reduction in virus production with ZAP expression.	Not measured	Not measured	[114]
***Flaviviridae***	*Japanese encephalitis virus (JEV)*	- Blocked by ZAP overexpression; - ZAP targets the 3′ UTR of JEV; - Hampered JEV translation.	- Exosome	- RIG-I signaling pathway	[10]
	*Zika virus (ZIKV)*	- Infection attenuated in vivo e in vitro; - Mutant viruses with frequencies of CpG or UpA dinucleotides showed attenuation of replication in vertebrate cell lines, which was rescued by knockout of ZAP; - ZIKV CpG- or UpA-high in mice did not cause typical ZIKV-induced tissue damage.	Not measured	Not measured	[26,115]
***Filoviridae***	*Ebola virus (EBOV)*	- Reduced the mRNA, inhibiting replication; - Motifs ZnF2 and ZnF4 needed to be intact for this antiviral activity of ZAP; - All isoforms (ZAP-L, ZAP-XL, ZAP-S, ZAP-M) acted similarly in inhibition of viral transcription and replication.	TRIM25	Type I IFN	[6,12,16]
	*Marburg virus (MARV)*	- Reduced the mRNA, inhibiting replication; - Motifs ZnF2 and ZnF4 needed to be intact for this antiviral activity of ZAP.	Not measured	Not measured	[6]
***Picornaviridae***	*Coxsackievirus B3*	- Animals more resistant to viral replication and myocarditis induction; - Prevented viral replication in vitro.	Not measured	Not measured	[14]
	*Echovirus 7 (E7)*	- Decrease in virus replication; - The bases 39 and 59 of CpG motifs influenced replication and ZAP binding.	- oligoadenylate synthetase 3 (OAS3)/RNase L	Not measured	[60,89,116]
	*Enterovirus A71 (EV-A71)*	- Infection increased the levels of ZAP-L mRNA.	Not measured	Not measured	[117]
	*Poliovirus*	- Increasing frequencies of CpG dinucleotides; there was a decreased replicative fitness in HeLa cells, as well as a reduction in poliovirus infectivity.	Not measured	Not measured	[118]
***Poxviridae***	*Vaccinia virus Ankara (MVA)*	- Limits MVA	Not measured	Not measured	[119]
***Hepadnaviridae***	*Hepatitis B virus*	- In vitro, overexpression of ZAP was able to control viral replication; - The N-terminal domain was important for this antiviral activity; - In vivo, HBV DNA replication intermediates were decreased by ZAP protein; - ZAP-L and ZAP-XL have an efficiency greater as an antiviral activity when compared to ZAP-S and ZAP-M.	Not measured	Not measured	[16,31,120]
***Hepeviridae***	*Hepatitis E virus*	- Inhibition of ZAP expression in patients with HEV genotype four acute infection; - ZAP overexpression inhibited HEV replication.	Not measured	IRF3 signaling	[121]
***Herpesviridae***	*Murine gammaherpesvirus 68*	- Binds to M2 mRNA (gene expressed in the latency phase of MHV-68) and leads to a decrease in its expression.	Not measured	Not measured	[33]
	*Human cytomegalovirus (HCMV)*	- ZAP-S inhibits replication; - HCMV evades ZAP - Detection through suppression of CpG dinucleotides; - ZAP-S and ZAP-L can inhibit expression of HCMV genes.	TRIM25	RIG-I and IRF3 signaling	[70]
***Coronaviridae***	*Severe acute respiratory syndrome* Enterovirus A71 *s 2 (SARS-CoV-2)*	- ZAP-L is more efficient in reducing SARS-CoV-2 RNA expression than ZAP-S; - ZAP-L requires the CaaX box to inhibit SARS-CoV-2 replication; - Restricted SARS-CoV-2 replication in vitro, particularly upon treatment with IFN-α or IFN-γ.	TRIM25KHNYN	Type I and II IFN	[24,25,51,122]
***Paramyxoviridae***	*Sendai virus*	- ZAP isoforms in humans do not differ in stimulating the expression of IFN type I; - hZAP mRNA was upregulated.	Not measured	Type I IFN; IRF3	[11,16]
	*Small ruminant morbillivirus (SRMV)*	- Inhibited replication in cells; - Overexpression of ZAP in Vero-SLAM cells significantly increased their resistance to SRMV replication.	Not measured	Not measured	[123]

## Data Availability

Not applicable.
